# Enabling nurses’ engagement in the design of healthcare technology – Core competencies and requirements: A qualitative study

**DOI:** 10.1016/j.ijnsa.2023.100170

**Published:** 2023-12-06

**Authors:** Thijs van Houwelingen, Alexandra C.M. Meeuse, Helianthe S.M. Kort

**Affiliations:** a University of Applied Sciences Utrecht, the Netherlands; b Eindhoven University of Technology, the Netherlands

**Keywords:** Biomedical technology, Stakeholder participation, Equipment design, Professional competence, Quality improvement, Intersectoral collaboration

## Abstract

**Background:**

Due to the globally increasing demand for care, innovation is important to maintain quality, safety, effectiveness, patient sensitivity, and outcome orientation. Health care technologies could be a solution to innovate, maintain, or improve the quality of care and simultaneously decrease nurses’ workload. Currently, nurses are rarely involved in the design of health care technologies, mostly due to time constraints with clinical nursing responsibilities and limited exposure to technology and design disciplines. To ensure that health care technologies fit into nurses’ core and routine practice, nurses should be actively involved in the design process.

**Objective:**

The aim of the present study was to explore the main requirements for nurses’ active participation in the design of health care technologies.

**Design:**

An exploratory descriptive qualitative design was used which helps to both understand and describe a phenomenon.

**Participants:**

Twelve nurses from three academic hospitals in the Netherlands participated in this study.

**Method:**

Data were collected from semistructured interviews with hospital nurses experienced in design programs and thematically analysed.

**Results:**

Four themes were identified concerning the main requirements for nurses to participate in the design of health care technologies: (1) nurses’ motivations to participate, (2) the process of technology development, (3) required competence to participate (such as assertiveness, creative thinking, problem solving skills), and (4) facilitating and organizing nurses’ participation.

**Conclusion:**

Nurses experience their involvement in the design process as essential, distinctive, and meaningful but experience few possibilities to combine this work with their current workload, flows, routines, and requirements. To participate in the design of health care technologies nurses need motivation and specific competencies. Organizations should facilitate time for nurses to acquire the required competencies and to be intentionally involved in technology design and development activities.

**What is already known**

• Health care technologies such as electronic health record systems do not always contribute to patient care because they are time-consuming or difficult to use.
• Although nurses, as end users, could have a valuable role in increasing the adoption of health care technologies, nurses are rarely involved in the actual design of health care technologies.
• There is a lack of knowledge about how hospital nurses can actively be involved in the design process and subsequently examine their role as quality promoters and innovators.

Alt-text: Unlabelled box

**What this paper adds**

• This paper suggests 39 competencies that are relevant for nurses to participate in health care technology design, which can be taught during nursing school, workshops, or specific minor or bachelor health care technology design training
• Designing should occur in cocreation because the nurses’ insights are necessary for the design of a solution related to a problem that was indicated from practice.
• Facilitating nurses in the health care technology design process requires organizational policy changes to make it possible for the organization to allow nurses to participate in the health care technology design process.

Alt-text: Unlabelled box

## 1 Introduction

The worldwide population is growing, people are getting older, and the demand for complex care increases (Nations U, 2017; Statistics Netherlands CBS, 2021). In the Netherlands, with the increasing number of patients, fewer nurses are available per patient to provide all patients' the care they need (Statistics Netherland CBS, 2019; Dutch Nurses’ Association, 2017). Because of the shortage of nurses, nurses have a high administrative burden and have little time to spend with their patients (Dutch Nurses’ Association, 2017; Myny et al., 2011; Mittmann et al., 2008; Lindqvist et al., 2014; Hinno et al., 2011). The less time nurses have to spend on patients, the lower the quality of care according to humanistic care theory (Dutch Nurses’ Association, 2017; Hinno et al., 2011; Bichel-Findlay et al., 2009; Braaf et al., 2011; Delmas et al., 2018; du Plessis, 2016; Khademi et al., 2017; Liu et al., 2018; Kraljic et al., 2017; Hurst, 2008). Quality of care is an important topic in the health care sector and should be safe, effective, patient-centered, and timely (Joshi et al., 2018; Baker, 2001).

High workload is experienced by all Dutch nurses, of which 40 % are hospital nurses (Liu et al., 2018; Statistics Netherlands CBS, 2018). To improve the quality of care by reducing nurses’ administrative tasks to create more time for patient contact, hospitals often use health care technologies. Examples of these health care technologies are electronic health record systems, robots, or measurement devices (Li and Benton, 2006; Bates et al., 2003; Jha et al., 2009). With correct and sufficient use of health care technologies, administrative burden could decrease, and errors could be recognized earlier (Bates et al., 2003; Baumann et al., 2018; Lee et al., 2018). Then, nurses would have more time to spend on the patients’ actual needs, and the quality of care could increase (Delmas et al., 2018; du Plessis, 2016; Khademi et al., 2017). Although health care technologies such as electronic health record systems exist, they do not always achieve their desired effect in helping reduce administrative tasks, but are instead taking up more of nurses time since they can be time-consuming or difficult to use (Baumann et al., 2018; Sharman, 2007). When health care technologies are adequately designed and aligned with nurses' daily practice, nurses can take advantage of the benefits of the use of technologies (Baumann et al., 2018; Drach-Zahavy et al., 2014).

Decisions about whether to use health care technologies are often made by hospital policy-makers and hospital management. The actual design of such health care technologies is mostly performed by engineers (Jha et al., 2009; Sharman, 2007; Chiang et al., 2017). Although nurses, as end users, could have a valuable role in increasing the adoption of health care technologies, nurses are rarely involved (Wyszewianski, 2018; Bolton et al., 2008). This lack of involvement could be seen as a missed opportunity, as nurses at wards are experts in delivering care and have the most adequate insights into what technologies are needed and what is needed to make technological innovations work effectively (Bates et al., 2003; Lopez et al., 2019). Currently, involving end users in design programs in the health care sector has become more common (Calvillo-Arbizu et al., 2019). The literature shows that involving end users, such as patients or health care professionals, in the health care sector is necessary and should occur during the entire process (Årsand and Demiris, 2008). Although nurses and designers both see the importance of the involvement of nurses in designing health care technologies in cocreation, the literature indicates that it is hard to involve them (Bolton et al., 2008; Lopez et al., 2019; Calvillo-Arbizu et al., 2019; Hamer, 2013). The lack of involvement could be caused by the limited time nurses have to perform their core nursing activities and their lack of leadership skills to defend their involvement rights and necessity. Additionally, knowledge of health care technologies (Hamer, 2013) could be lacking.

Hospital nurses should actively engage in the design of new health care technologies to guarantee that they are in line with routine nursing practice and have the potential to enhance the quality of care (Calvillo-Arbizu et al., 2019; Årsand and Demiris, 2008; Hamer, 2013). Although the literature shows what competencies (blend of knowledge, skills and attitudes) and knowledge nurses might need to use technology in practice and how to design (van Houwelingen et al., 2016; Steeringgroup Bachelor of Nursing, 2015), there is a lack of knowledge about how hospital nurses can actively be involved in the design process and subsequently examine their role as quality promoters and innovators (Marques et al., 2011). Gaining a deeper understanding of nurses' involvement in health care technology design could help identify the fundamental requirements and competencies (knowledge, skills and attitudes) needed for this position. This study aims to explore what the main requirements are to facilitate hospital nurses to actively participate in the design of health care technologies.

## 2 Method

### 2.1 Design

An exploratory descriptive qualitative design was used (Polit and Beck, 2017; Cooper and Endacott, 2007). An exploratory descriptive design helps to both understand and describe a phenomenon that has not been studied in-depth in prior research (Hunter et al., 2019).

### 2.2 Population and setting

The study was conducted in three academic hospitals in the Netherlands. Participants were included if they (1) could speak Dutch fluently (native Dutch), (2) had graduated and were registered hospital nurses, (3) had participated at least once in a design project for a health care technology to reflect on the requirements to participate based on their experience, and (4) had at least once worked with health care technologies in practice. Recruitment of participants was done by purposive sampling to come to a heterogeneous sample with variance in gender, age, and work experience for representativeness to other hospital nurses (Holloway and Wheeler, 2014).

### 2.3 Data collection & procedures

Recruitment of study participants occurred between February and March 2019. Potential participants known by the researchers involved in this project network were invited by email with an information letter about the study. Participants were allowed to forward the invitation in their network to recruit other participants. When participants were interested in participating and met the inclusion criteria, they were asked to send the researcher their approval by email. Then, the interview was planned, and further study information was sent to the participant.

Semistructured interviews were used to gather data since they allowed for the systematic exploration of particular topic areas of interest (Holloway and Wheeler, 2014). Interviews were conducted by the second author, who is a master's student in clinical health science. To ensure quality, before the interviews, the second author was trained in interview techniques. The content of the interview guide was validated in accordance with Artino et al. (2014) guidelines. Outcomes of previous research were used to formulate topics, e.g., role as co-designer, required knowledge, skills, attitudes, technological experience, phases of involvement in the design process (Lopez et al., 2019; Hamer, 2013). By discussing the interview guide with an expert panel of two independent researchers with experience in the research topic and with the research team, the guide was further validated to enhance quality.

Before the actual interviews, a pilot interview was conducted to establish whether questions and topics in the guide were relevant and interpreted adequately. The pilot interview was held with a recently registered nurse. By evaluating the results of the pilot interview, no questions were added or removed. Each interview was audio-recorded in the nurses’ workplace at the hospital in a room with limited ambient noise. By fostering a nonjudgmental environment throughout the semi-structured interviews to hear the participants' perspectives, the researcher built the study's reliability. The interviews lasted from 30 minutes to 1 hour. Before the interviews started, participant demographics such as age and work experience were collected. In every interview, memos of impressions and remarks were made. Interviews were held concurrently with data analysis and stopped when data saturation was achieved.

### 2.4 Data analysis

To analyse the data, the thematic analysis method by Braun and Clarke (2012). The interviews were transcribed verbatim in Microsoft Word 2016. Verbatim transcription of the interview results lowers the possibility of bias. First, transcripts were read and reread to become familiar with the data. Thereafter, in phase two, initial codes were generated. Therefore, the first transcript was coded together by two of the authors to reach an agreement about coding meaningful parts of the data using NVIVO version 12, QRS international software.

Themes were searched by categorizing codes by relevance and relation to each other. These categories of codes were then further organized into more meaningful themes and were reviewed with the data. Finally, in the last phase, the identified themes could be defined and named. To ensure quality, the defined themes were discussed with all authors, and together, we agreed on the themes. Finally, the themes were submitted for member checking (Bygstad and Munkvold, 2007) by sending them to three participants, each from a different hospital to ensure quality. Peer review at researcher meetings and investigator triangulation during data processing increased the conformability of the interpretation and credibility of the findings, hence enhancing the study's trustworthiness.

### 2.5 Post hoc analysis

Since participants mentioned all kinds of competencies as being essential for nurses to be able to effectively participate in the design process, and some competencies were mentioned only once, whereas some were mentioned more often, we performed a post hoc analysis to further validate these competencies. In line with the content validity method developed by Lynn (1986), we asked participants to review a list with all competencies that were mentioned and indicate the relevance of each item using a 4-point Likert scale: 1=very irrelevant, 2=irrelevant, 3=relevant, and 4=very relevant.

Based on the scores, a relevance score for each competency was calculated as follows. First, the scores were dichotomized (0 = very irrelevant and irrelevant, 1 = relevant and very relevant). Second, the score for each competency was divided by the number of participants. In line with the content validity method described by Lynn (1986), we used a relevance threshold of 78 %; only items with a score ≥0.78 were considered ‘relevant’.

### 2.6 Ethical issues

This study was conducted following the principles of the Declaration of Helsinki (World Medical Association, 2013). Because participants did not undergo any form of treatment or action, the Dutch Medical Research Involving Human Subjects Act did not apply. Therefore, no official ethical approval was required (Central Committee on Research Involving Human Subjects, 2020). At the beginning of each interview, informed consent was signed by the participants and the PR. Data were stored confidentially by numbering participants, transcripts, and audio data, with access only by the researchers. After publication, the data will be kept for 15 years.

## 3 Results

### 3.1 Demographics

In total, twelve hospital nurses participated in this study, of which nine were female. The ages ranged from 28 to 59 years, with a working experience range of 7 to 35 years. Saturation of data was achieved by twelve participants when no more new information was mentioned.

### 3.2 Main themes

From the initial data analysis, 300 codes emerged, which were subsequently clustered into four main themes: (1) nurses’ motivation to participate, (2) process of technology development, (3) required competence to participate, and (4) facilitating and organizing nurses’ participation. All four main themes were mentioned by all participants. Below, the four themes are described and illustrated with exemplary quotes.

### 3.3 Nurses’ motivation to participate

To be involved early in the design process, most participants mentioned that nurses had to be motivated. Nurses’ motivation seemed to be built on experiences and their understanding of the importance of their participation in design processes. Nevertheless, according to the participants, nurses know best what happens in practice and which health care technologies are necessary. Participants mentioned that working with health care technologies is a part of the job, and technologies will be more adopted in the future. According to participants, most nurses are interested in health care technologies and enjoy working with health care technologies. Participants described health care technologies as necessary to provide quality care. They believe that participating in the health care technology design process contributes to their self-esteem and will give them confidence.

“*That something you have designed and produced in here which is produced thereafter, how proud can you be about yourself and your nursing profession.”* (P1)

However, not all nurses appeared to be motivated to participate in the health care technology design process. Resistance to health care technology development and implementation arises when nurses believe that using health care technologies is difficult and hard to understand or health care technologies do not interest them. According to some participants, nurses’ professional language differs from the language of health care technology developers. Language barriers and disparate jargons can cause misunderstandings in interdisciplinary collaboration, so participants stress the value of effective communication and the necessity of learning each other's language in order to collaborate successfully.

“*I find it striking that someone speaks in a different language. Initially, I needed to get used to it. "Why don't they [technicians] understand that," I asked myself.”* (P2)

Additionally, they conceptualize things in another manner. Therefore, it could be challenging for nurses to be motivated. Finally, the participants mentioned that the importance of involving nurses was not even considered, which demotivates them to take initiative to participate.

“*It seems that the importance of involving a nurse is not sufficiently taken into account in this.”* (P7)

### 3.4 Process of technology development

Most participants mentioned that the involvement of nurses in the health care technology design process should begin at the start of the process when discussing the needs and every single problem that needs to be solved. Then, according to the participants, the involvement of nurses is also required during the development phase, piloting, implementing health care technologies, and evaluating the designed health care technologies.

“*Nurses are almost always the end user of a technology. Thus, it should come as normal that nurses have a seat at the table. So, as well during the design stage and not only during the execution*.” (P6)

Participants mentioned that nurses have insights into the way existing health care technologies work in practice, what health care technologies are missing in daily practice, or what the main problem of existing health care technologies is. Therefore, participants experience their involvement as end users in the development process as necessary.

“*I think, it is a very missed opportunity, if you want to introduce something new and there are no nurses involved.”* (P1)

However, several participants mentioned that not all nurses needed to participate in the entire health care technology design process. These participants suggested that nurses should only be able to suggest ideas or give feedback on the technologies that are already designed.

Participants indicated that designing health care technologies should be done in cocreation in good collaboration with a variety of disciplines. Therefore,working in cocreation means that also nurses' feedback should be taken into account.

“*If you want to develop technology for the benefit of patient care, you also need those [nurses] people.”* (P8)

Nevertheless, participants mentioned that the process of designing, implementing, and innovating takes considerable time and is hard. Therefore, participants suggested that designing must be accessible and include several rounds discussing small problems that need to be solved. Then, during the process, nurses should be assisted by coaches for a better understanding of the design process.

### 3.5 Required competencies to participate in design

To participate in designing projects, nurses need several competencies. Initially, a total of 48 competencies were mentioned by the participants. Eleven participants (of 12) participated in the post hoc analysis. After the post hoc analysis, using a relevance threshold of 78 %, 39 competencies remained. These competencies could be divided into three subthemes: knowledge, skills, and attitudes. All 39 competencies are listed in Table 1.

**Table 1:** Essential competencies for nurses to participate in the design of health care technologies.

Competencies	Relevance Score
***Knowledge***	
1. Insight into the nurses workflow	1,00
2. Insight into the required and necessary skills	0,91
3. Knowledge of possibilities within the organization	0,91
4. Insight into development process	0,82
5. Knowledge of technology	0,82
***Skills***	
1. Being able to rethink how things could be done	1,00
2. Being able to communicate well	1,00
3. Transfer enthusiasm	1,00
4. Translate innovations and solutions to own practice	1,00
5. Being practical in finding solutions	1,00
6. Estimate what works in practice and what doesn't	1,00
7. Turning signals into the practice	1,00
8. Being innovative	0,91
9. Being able to switch quickly between subjects	0,91
10. Being able to coordinate projects and wards	0,82
11. Creative thinking	0,82
12. Thinking out-of-the-box	0,82
13. Transcendent thinking	0,82
14. Using persuasiveness	0,82
15. Project management skills	0,82
16. Having work experience before participating	0,82
***Attitudes***	
1. Being critical	1,00
2. Being intrinsically motivated	1,00
3. Showing perseverance	1,00
4. Taking initiative	1,00
5. Being enthusiastic	1,00
6. Taking an advisory role	1,00
7. Being curious	1,00
8. Being open to innovation	1,00
9. Being proactive	1,00
10. Being tenacious	1,00
11. Taking responsibility and showing professionalism	1,00
12. Demonstrating and using nursing leadership	1,00
13. Being visible	1,00
14. Having courage	0,91
15. Being flexible	0,91
16. Being open to making mistakes	0,91
17. Being proud to be a nurse	0,91
18. Taking on a design role	0,82

*Note:* Items arranged from high to low *within each category (Knowledge, Skills, Attitudes).* The relevance score was dichotomized (0 = very irrelevant and irrelevant, 1 = relevant and very relevant). The scores above were calculated by dividing the score for one item by the number of participants. Subsequently, 1 is the maximum score. In this study, only items with a score ≥0.78 were considered ‘relevant’, in line with the content validity method by Lynn (1986). Items with a score <0.78 are not listed in the Table.

#### 3.5.1 Knowledge

Participants indicated that nurses must possess 5 types of knowledge. They need to have insights and knowledge about daily nursing practices and what is necessary to make health care technologies work in their daily practice. According to the participants, knowledge about how technologies are designed, and knowledge of practice is also required to understand and be able to talk and think about designing new health care technologies. Furthermore, participants indicated that nurses needed to know their work network connections well.

*“As a nurse you naturally have a view on what works and what does not work, or whether it fits in the workflow.”* (P1)

#### 3.5.2 Skills

Participants identified 16 skills required to participate in the health care technology design process. Many participants indicated you have to be able to transfer enthusiasm to recruit colleagues during the process and convince others of the necessity of new health care technologies. Participants also noted that it is important to be skilled in communication with other disciplines and how to communicate in groups to translate outcomes of health care technology designs into practice. Another important skill noted by participants is that you must be able to think out of the box, think in creative solutions, be able to work in project groups, and be able to think transcendently.

“*You must dare to look beyond the boundaries of the project itself.”* (P11)

#### 3.5.3 Attitudes

When nurses want to participate actively in the design of health care technologies, 18 different attitudes are required. There was 100 % consensus about the relevance of the first 13 attitudes listed in Table 1, e.g., being enthusiastic, being intrinsically motivated, being curious, and being critical. Furthermore, nurses should have courage and flexibility.

“*You must be able to be critical and make good judgments.”* (P7)

#### 3.5.4 Education

According to most participants, nurses have to be educated in competencies such as knowledge, skills, and attitudes to be able to participate in the health care technology design process. Therefore, participants noted that education in these competencies should be part of the undergraduate nursing curriculum. Participants recommended that nursing schools educate future nurses in these skills by providing an assignment to practice these skills and become educated in them.

“*Bachelor nursing students have to do an innovation assignment; I think that is a very good idea.”* (P12)

Participants mentioned possibilities to get graduate nurses skilled in competencies by participating in further pieces of training and workshops. Another possibility mentioned by participants is to train future and graduate nurses in a separate, specific course or even a complete, specific bachelor's program about innovating health care technologies.

### 3.6 Facilitating and organizing nurses’ participation

Participants noted that nurses had to be facilitated in actually applying their innovative skills in practice. Although all nurses should have the opportunity to participate in the health care technology design process, the role of a participating nurse in a health care technology design program is important. As a participating nurse, you are the focal point for colleagues on the ward and the center point of patient care. In this role, nurses use the input about questions and problems with health care technologies in daily practice that they receive from the working groups and team meetings on the wards .

To make it possible for nurses to participate and give input for health care technology innovations, participants mentioned that hospital management could organize innovation meetings and contests to motivate and encourage nurses.

*“We also have some innovation days, as a nurse you can come and say: I have an idea, what do you think of it?”* (P9)

Participants mentioned the Dutch innovation centers, *Create4Care* in Erasmus MC Rotterdam and *Reshape* in Radboud MC in Nijmegen, as initiatives other organizations could use as an example. In these facilities, nurses can walk in, participate and start designing health care technology solutions by themselves with the help of coaches. As a result, participants noted that nurses should be given time to participate in these meetings during work hours. If nurses do not participate actively in the health care technology design process, participants said that this participation could be stimulated by personally inviting nurses.

*“Every organization should actually require that there be a nurse in every working group that is set up that deals with healthcare. Nurses must show initiative in this, but can also be held accountable for their responsibility within innovation. On the one hand, by asking directly or on the one hand by simply putting people in a working group, otherwise that change will not happen.”* (P4)

Participants also mentioned that when they were skilled and wanted to participate, they did not always receive permission. Team managers want nurses to stay in wards because there is a shortage of nurses. Patient care is prioritized, and consequently, no time can be freed to participate in the health care technology design process. Participants mentioned that their organization should set a clear mission and vision about the usage and development of health care technologies by all disciplines. Therefore, participation in the health care technology design process should be the subject of discussion at wards and in hospitals. Team managers should give their approval when nurses want to participate. Designing and innovating health care technologies is a part of the job.

*“It just has to be self-evident that you are there [in the project group], that innovation is also part of your nursing work.”* (P8)

## 4 Discussion

This study identified four main themes for the requirements of nurses to participate actively in the health care technology design process. First, nurses should be motivated and enthusiastic. Therefore, nurses need to be assured that their input will be used. Second, designing should occur in cocreation because the nurses’ insights are necessary for the design of a solution related to a problem that was indicated from practice. The entire design process must be accessible for all nurses, during which they should get help from coaches. Third, nurses need 39 competencies to participate in health care technology design, which can be taught during nursing school, workshops, or specific minor or bachelor health care technology design training. Fourth, hospitals have to facilitate and organize the opportunity for nurses to participate in the health care technology design process, such as facilitating time. Therefore, hospital management should set a clear policy and vision about how to innovate health care.

### 4.1 Comparison with prior work

Prior research shows that there are eight essential competencies in which hospital nurses should be educated in how to use health care technologies in practice (van Houwelingen et al., 2016). Currently, Dutch bachelor nurses are already becoming educated in CanMEDS roles in the newest curriculum (Steeringgroup Bachelor of Nursing, 2015). The CanMEDS roles describe that nurses have to be educated in technology as a professional and quality improver (Steeringgroup Bachelor of Nursing, 2015). The current study identified which competencies nurses need to actively participate in the design of health care technologies to improve health care. This knowledge is an addition to existing knowledge.

To strengthen nurses in their role as codesigners, the present study states that nurses need to be assertive and show nursing leadership. Nursing leadership is also one of the CanMEDS roles in which new graduate nurses are educated (Steeringgroup Bachelor of Nursing, 2015). The literature shows that nurses need these nursing leadership skills to have an active role in the development of new policies or new health care technologies (AbuAlRub and Foudeh, 2017; Burkoski, 2019). Nursing leaders are essential for motivating and have an important role in distinguishing whether health care technologies will be implemented to provide high quality care (Hamer, 2013; Burkoski, 2019; Castner et al., 2016).

The role of nurses in the design process is comparable to the role of nurse scientists who aim to close the gap between scientific knowledge and clinical practice, otherwise known as “knowledge brokers” (Thompson et al., 2019) or “boundary spanners” (McNiven et al., 2021). In both roles, nurses employ ‘boundary spanning activities’ in which nurse scientists bridge science with clinical practice and nurses who participate in the design process bridge the domain of technology with clinical practice.

The current study shows the necessity of nurses’ involvement in the health care technology design process. Prior research determined outcomes in line with these outcomes (Suserud et al., 2013). When health care technologies are developed adequately, it increases quality of care and increases work satisfaction. The facilitation of nursing participation in the health care technology design process is complicated, as shown in this study. This complexity is due to hospital policy and nonpatient time, which nurses are allowed to use, which is also shown in the literature (AbuAlRub and Foudeh, 2017).

### 4.2 Limitations and strengths

The inclusion of a participant who did not meet all inclusion criteria was a limitation of the study. The lack of experience with the health care technology design process made it impossible to explore the experiences in line with the research question. However, these data were analyzed in line with the other data. To prevent bias, these divergent data should have been analyzed separately from the other data to compare the outcomes. Another limitation is discussing topics such as competencies and facilities that might have affected participants' responses by making the participants think in a certain direction. However, prior studies determined these topics as relevant to include (Lopez et al., 2019; Hamer, 2013).

Furthermore, the use of a valid method for developing an interview guide is a strength of this study to ensure quality (Artino et al., 2014). Because an expert panel of independent researchers discussed and validated the interview guide, some necessary alterations were made. Finally, the data analysis was executed by two authors, which strengthens the study. Investigator triangulation reduces the risk of biased decisions about coding relevant data and defining themes. By analysing and discussing the data, the most relevant data were coded, and defined themes were agreed upon by all researchers.

### 4.3 Implications for practice, education, and future research

Nurses who are intrinsically motivated to participate in the health care technology design process should be proactive and start conversations with their team and managers about participation. Therefore, when the hospital is innovating, nurses should show their courage and search for knowledge about how they could participate. Then, nurses should just start by taking part in meetings about innovating health care to show their experiences and input. Design teams should allow nurses to explain the necessity of nursing involvement and input in the health care technology design process. When nurses have not invited themselves, the design teams should actively search for motivated nurses and invite them to participate in the health care technology design process.

There are different ways and examples of how nurses can contribute to the development and implementation of technological innovations. Schleyer (2022) provides a detailed description of the Chief Nursing Informatics Officer (CNIO) position as one example. CNIOs hold a specific role in bridging the clinical care and IT domains. CNIOs can share technical opportunities and insights with clinical professionals and represent the nursing and interprofessional clinical perspective to IT.

However, nurses can also take part in the design of an innovation for the duration of a single project. Rigtering et al. (2023) performed an in-depth case study of Create4Care, a medical makerspace of the largest academic hospital in the Netherlands. Patients, caregivers, and nurses can bring ideas for improvements or issues they are facing to Create4Care. Rigtering et al. conclude that developing an innovation ecosystem is crucial for the diffusion of nurse innovations. This ecosystem can be seen as a setting where each stakeholder e.g., students, patients, nurses, and professionals developing the innovations) contributes in a way that these actors achieve complementary benefits. In such way nurses can get aligned with design teams and developers. The researchers describe that most nurses do not continue with the further development of an innovation because they anticipate that it will be too time-consuming and challenging to turn an early solution into an innovation that can be shared with others. A medical makerspace, like Create4Care, effectively tackles this issue by using their innovation ecosystem that primarily manages the process of invention and diffusion.

Additionally Barr et al. (2021), explored the experiences with nurse innovation centers in the United States and found six themes that are important for the sustainability and impact of nursing innovation centers: funding, engagement, leadership, interprofessional collaboration, diversity and partnerships. One of the researcher's recommendations is to create educational opportunities to prepare nurses to innovate. The 39 competencies identified in this study can serve as a foundation for the design of educational and training programs. To obtain nurses educated in the required competencies, it is recommended that nursing schools incorporate and increase knowledge of designing health care technologies in their curriculum. Additionally, a special educational program for designing health care technologies could be developed, nurses in practice.

Above, based on this study results and what is known from the literature, we outline how nurses can engage in the design of technology and the ways nursing schools can expedite this by providing nurses with the necessary competencies. However, facilitating nurses in the health care technology design process requires organizational policy changes to make this to happen. Hospital management should allow motivated nurses to first participate in the health care technology design process as ambassadors. They should facilitate them in using time during working hours. In addition, hospital management should discuss their vision about innovating care to ensure innovation is the main goal. Then, the hospital policy could be described with this vision, which incorporates involving all end-users in the health care technology design process.

To encourage hospital boards in this direction, further research is recommended to gain insight in the impact of the actively involving nurses in the design of technology and how this benefits to quality improvement and job satisfaction.

## 5 Conclusion

To participate actively in the health care technology design process, there are four main requirements for hospital nurses. Nurses need to be motivated and enthusiastic. Therefore, the design process has to occur in cocreation to use the experiences and input of the end-users. Required competencies to participate are knowledge, skills, and attitudes of health care technologies that could be improved. When hospital organizations facilitate nurses in time and facilities to participate in the health care technology design process, which might eventually lead to more adequately designed health care technology solutions that can help to improve quality of care.

## Funding

This project was funded by SIA Raak Publiek (RAAK.PUB04.008). The funding organization had no input into the planning, execution, analysis, interpretation, or writing of the study's data or manuscript.

## CRediT authorship contribution statement

**Thijs van Houwelingen:** Conceptualization, Methodology, Project administration, Investigation, Formal analysis, Writing – review & editing, Supervision. **Alexandra C.M. Meeuse:** Writing – original draft, Conceptualization, Methodology, Investigation, Formal analysis. **Helianthe S.M. Kort:** Conceptualization, Methodology, Supervision, Writing – review & editing.

## Declaration of competing interest

None.
